# Three new species of *Russula* subsect. *Cyanoxanthinae* from subtropical China (Russulales, Russulaceae)

**DOI:** 10.3897/mycokeys.134.195117

**Published:** 2026-06-23

**Authors:** Ben-Jian Zhong, Hui Zeng, Sheng-Nan Wang, Jun-Qing Yan

**Affiliations:** 1 Jiangxi Provincial Key Laboratory of Excavation and Utilization of Agricultural Microorganisms, Jiangxi Agricultural University, Nanchang 330045, China Jiangxi Provincial Key Laboratory of Excavation and Utilization of Agricultural Microorganisms, Jiangxi Agricultural University Nanchang China; 2 Institute of Edible mushroom, Fujian Academy of Agricultural Sciences, Fuzhou 350011, China Jiangxi Provincial Key Laboratory of Subtropical Forest Resource Cultivation, College of Forestry, Jiangxi Agricultural University Nanchang China; 3 Jiangxi Provincial Key Laboratory of Subtropical Forest Resource Cultivation, College of Forestry, Jiangxi Agricultural University, Nanchang 330045, China Institute of Edible mushroom, Fujian Academy of Agricultural Sciences Fuzhou China

**Keywords:** Ectomycorrhizal fungi, *
Heterophyllidiae
*, multi-locus phylogeny, novel species, taxonomy

## Abstract

Three new species belonging to *Russula* subsect. *Cyanoxanthinae*, namely *R. purpureogrisea*, *R. subpallescens*, and *R. wuyishanensis*, are described from subtropical China based on combined morphological and molecular evidence. Morphologically, *R. purpureogrisea* is distinguished by a dark greyish-ruby to greyish-brown pileus, close lamellae with lamellulae, a white context that remains unchanging when bruised, and basidiospores with ornamentation composed mostly of obtuse-conical isolated warts, occasionally connected by fine lines or low ridges, occasionally forming a subreticulum locally. *Russula subpallescens* is recognized by a pinkish-white pileus, adnate lamellae of equal length, an unchanging context, a white stipe that develops pale yellow to maize-yellow patches with age or long after bruising, and basidiospores with ornamentation composed of dispersed warts, frequently connected by short lines, nearly subreticulate. *Russula wuyishanensis* features a variable pileus color ranging from reddish-grey to purplish-brown, three types of lamellulae, a white stipe occasionally with rust-colored patches, a white context turning yellowish-brown when bruised, and basidiospores with ornamentation predominantly consisting of isolated warts, partially connected by short lines or ridges, occasionally subreticulate. The independent taxonomic status of these three novel taxa is strongly supported by multi-locus phylogenetic analyses based on four gene regions (ITS, LSU, mtSSU, and *rpb*2). Detailed descriptions and morphological photographs of the new species are provided herein.

## Introduction

*Russula* Pers. (Basidiomycota, Agaricomycetes, Russulales, Russulaceae) is the type genus of Russulaceae, and one of the most species-rich fungal genera worldwide, with an estimated minimum of 2,000 accepted species (Hyde et al. 2024). It has a cosmopolitan distribution across diverse ecosystems, ranging from arctic tundra to tropical forests (Buyck et al. 2018; Adamčík et al. 2019; Wijayawardene et al. 2020). Species in this genus form widespread ectomycorrhizal (ECM) symbioses with a diverse array of host plants, encompassing gymnosperms, such as *Abies* and *Pinus*, alongside angiosperms, like Fagaceae and Fabaceae (Wang et al. 2024). Such mutualistic partnerships are crucial for bolstering plant stress tolerance, stimulating root proliferation, and improving nutrient acquisition, thereby fundamentally underpinning the vitality and sustainability of forest ecosystems (Looney et al. 2018; Sarwar et al. 2020). In addition, the genus holds high economic and pharmacological value, many species are popular wild edible mushrooms rich in nutrients, and have been confirmed to possess multiple important bioactivities, including anti-tumor, antioxidant, hypoglycemic, and hypolipidemic effects (Kaewnarin et al. 2016; Khatua et al. 2021a; Liu et al. 2023).

The vast species diversity and highly conserved macromorphology within the genus pose significant challenges to accurate species identification and the efficient utilization of its taxonomic resources. In recent years, advances in molecular systematics have provided a critical tool to address this long-standing problem. Based on the multi-gene phylogenetic framework, *Russula* is currently divided into nine subgenera, namely *Russula* subg. *Archaeae* Buyck and V. Hofst., *R.* subg. *Brevipedum* Buyck & V. Hofst., *R.* subg. *Compactae* (Fr.) Bon, *R.* subg. *Crassotunicatae* Buyck & V. Hofst., *R.* subg. *Cremeoochraceae* Buyck & X.H. Wang, *R.* subg. *Glutinosae* Buyck & X.H. Wang, *R.* subg. *Heterophyllidiae* Romagn., *R.* subg. *Malodorae* Buyck & V. Hofst., and *R.* subg. *Russula* Pers., in the mainstream global taxonomic system (Buyck et al. 2018; Buyck et al. 2020; Buyck et al. 2024). Among these subgenera, subsection *Cyanoxanthinae* Singer, an important lineage within subgenus *Heterophyllidiae* Romagn., is distinguished from other subsections by a unique set of diagnostic traits: lamellae frequently forked with occasional lamellulae, mild context taste, basidiospores usually with an inamyloid suprahilar spot, pileipellis showing metachromatic reaction in cresyl blue, and unicellular pileocystidia (Sarnari 1998; Buyck et al. 2018; Buyck et al. 2020; Buyck et al. 2024; Chen et al. 2024a).

As a key hotspot for the distribution and diversification of ectomycorrhizal fungi, the subtropical region of China features a warm and humid climate, complex and diverse landforms, and rich vegetation types dominated by evergreen broad-leaved forests, which provide highly favorable ecological conditions for the growth and reproduction of *Russula* species (Gao et al. 2013). In recent years, with the progress of in-depth macrofungal diversity surveys, Chinese mycologists have successively described at least 13 novel species in subsection *Cyanoxanthinae* (Zhao et al. 2015; Zhang et al. 2017; Li and Deng 2018; Song et al. 2019; Song 2022; Chen et al. 2024a, 2024b). Nevertheless, species in this subsection exhibit high phenotypic plasticity in macromorphology (e.g., extreme variation in pileus colour) and a widespread occurrence of cryptic species (Song et al. 2019; Chen et al. 2024a). Due to these challenges, early morphology-based identifications have masked numerous cryptic lineages, making many taxa highly prone to misidentification (Buyck et al. 2018). In particular, subtropical China, functioning as an ecological and evolutionary transition zone with complex landforms and high host-plant diversity (Gao et al. 2013), has historically lacked in-depth phylogenetic surveys. The recent discovery of a large number of novel species in provinces surrounding Jiangxi strongly indicates that the true phylogenetic diversity of this lineage in this critical region has been severely underestimated in the past (Zhang et al. 2017; Li and Deng 2018; Song 2022; Chen et al. 2024a, 2024b).

To this end, three previously unknown species belonging to subsection *Cyanoxanthinae* were discovered during recent field surveys of macrofungal diversity in this region. In the present study, we provide detailed morphological descriptions, comprehensive morphological comparisons, and molecular phylogenetic evidence for the formal taxonomic establishment of these three new species.

## Materials and methods

### Morphological studies

Specimens for this study were collected from Hubei and Fujian provinces, China, between 2022 and 2024. All specimens were dried at 50 °C and deposited in the Fungal Herbarium of Jiangxi Agricultural University (HFJAU). Fresh specimens were photographed and their macroscopic characteristics were recorded in the field. Color descriptions follow the “Methuen Handbook of Colour” (Kornerup and Wanscher 1978).

Microscopic structures were observed and measured using a LEICA DM2500 LED microscope (Leica Microsystems, Wetzlar, Germany). The description of microscopic features follows the template proposed by Adamčík et al. (2019). Briefly, sections of dried specimens were mounted in 5% KOH or water. A 1% Congo red solution was used as a stain to observe hyaline structures. Basidiospores were observed in Melzer’s reagent to test for amyloid reactions, and measurements were taken in lateral view, excluding surface ornamentation and the hilar appendix. The pileipellis was examined in cresyl blue to detect orthochromatic or metachromatic reactions (Buyck 1989). Sulphovanillin (SV) was used to observe color changes in the contents of cystidia (Caboň et al. 2017).

For each specimen, at least 40 basidiospores, 20 basidia, and 20 cystidia were measured. The size range of basidiospores is presented as (a) b–c (d), where “a” and “d” represent the extreme minimum and maximum values, respectively, and “b–c” encompasses 90% of the measured values. The abbreviation “Q” denotes the length/width ratio of the basidiospores in lateral view. A scanning electron microscope (SEM, JEOL JSM-IT800) was used to illustrate the structure and ornamentation of the basidiospores.

### DNA extraction, PCR amplification, and sequencing

Genomic DNA was extracted from dried specimens using the NuClean Plant Genomic DNA Kit (CWBIO, China) (Wang et al. 2022). Four loci, including ITS, LSU, mtSSU, and *rpb*2, were amplified and sequenced. The ITS region was amplified using the primer pair ITS1/ITS4 (White et al. 1990). The nLSU region was amplified using the primers LR0R and LR5 or LR7 (Vilgalys and Hester 1990). The *rpb*2 region was amplified using the primers fRPB2-6F/fRPB2-7cR (Matheny 2005). The mtSSU region was amplified using the primers MS1/MS2 (White et al. 1990).

PCR amplifications were performed in a 25 µL reaction volume containing 1 µL of DNA template, 1 µL of each forward and reverse primer, 9.5 µL of ddH_2_O, and 12.5 µL of 2× TaqMaster Mix (Wuhan Tsingke Biotechnology Co., Ltd., China). The PCR thermal cycling conditions for the four gene fragments were as follows: for ITS and nLSU, an initial denaturation at 94 °C for 5 min; followed by 35 cycles of denaturation at 94 °C for 50 s, annealing at 55 °C (for ITS) or 50 °C (for LSU) for 50 s, and extension at 72 °C for 1 min; and a final extension at 72 °C for 10 min. For mtSSU, an initial denaturation at 95 °C for 3 min; followed by 30 cycles of denaturation at 95 °C for 50 s, annealing at 55 °C for 30 s, and extension at 72 °C for 1 min; and a final extension at 72 °C for 10 min. For *rpb*2, an initial denaturation at 94 °C for 5 min; followed by 40 cycles of denaturation at 94 °C for 30 s, annealing at 52 °C for 40 s, and extension at 72 °C for 1 min; and a final extension at 72 °C for 10 min. The PCR products were sequenced by Qing Ke Biotechnology Co. Ltd. (Wuhan City, China).

### Alignment and phylogenetic analyses

A total of 174 sequences (54 ITS, 45 LSU, 38 *rpb*2, and 37 mtSSU) was used for phylogenetic analyses using Bayesian Inference (BI) and Maximum Likelihood (ML). The selection of sequences was based on the BLAST results of the ITS region and the studies by Song (2022) and Chen et al. (2024a) (Table 1). Following the analytical strategy of Chen et al. (2024a), *Russula maguanensis* and *R. substriata* were chosen as outgroups. The four gene datasets were independently aligned using the MAFFT online server with the auto-selection strategy (Katoh et al. 2019). The aligned sequences were manually trimmed at both ends using BioEdit (Hall 1999) and subsequently concatenated into a multi-locus matrix using PhyloSuite (Zhang et al. 2020). BI and ML phylogenetic analyses of the concatenated sequence dataset were performed using MrBayes v.3.2.7a (Ronquist et al. 2012) and IQ-TREE v.2.1.2 (Nguyen et al. 2015), respectively.

**Table 1.** Details of sequences used in the phylogenetic analyses. Newly generated sequences were in bold.

**Species**	**Voucher**	**Location**	**ITS**	**LSU**	***rpb*2**	**mtSSU**	**Reference**
*R. atrochermesina*	RITF6878 (holotype)	Yunnan, China	OR907106	OR907057	OR914538	OR934536	Chen et al. (2024a)
*R. atrochermesina*	RITF6460	Yunnan, China	OR907107	OR907056	–	OR934535	Chen et al. (2024a)
*R. banwatchanensis*	BBH 49228 (holotype)	Thailand	MT940813	MT965687	–	–	Crous et al. (2022)
*R. cyanoxantha*	UE29.09.2002-2	France	DQ422033	DQ422033	DQ421970	–	Buyck et al. (2008)
*R. cyanoxantha*	FH 12–201	Germany	KR364093	KR364225	KR364341	–	De Crop et al. (2017)
*R. cyanoxantha*	PC SM/BB 5	–	AY061669	–	–	–	–
*R. dinghuensis*	K15052704 (holotype)	Guangdong, China	KU863581	MK881922	–	MK882050	Zhang et al. (2017)
*R. flavobrunnea* var. *violaceotincta*	71/BB 06.050	Madagascar	–	KU237468	KU237754	KU237312	Buyck et al. (2018)
*R. fusiformata*	K15052703 (holotype)	Guangdong, China	MK049978	MK881942	–	MK882070	Song (2022)
*R. icterina*	RITF6080	Yunnan, China	PP700455	–	–	–	Chen et al. (2024b)
*R. icterina*	RITF6773	Zhejiang, China	PP700453	PP700463	PP707792	PP700445	Chen et al. (2024b)
*R. icterina*	RITF7066	Fujian, China	PP700454	PP700464	PP707791	PP700444	Chen et al. (2024b)
*R. icterina*	RITF6686 (holotype)	Jiangsu, China	PP700452	PP700462	PP707790	PP700443	Chen et al. (2024b)
*R. lakhanpalii*	RITF6474	Yunnan, China	OR907092	OR907077	OR914544	OR934527	Chen et al. (2024a)
*R. lakhanpalii*	RITF6868	Yunnan, China	OR907091	OR907078	OR914543	OR934528	Chen et al. (2024a)
*R. lakhanpalii*	CAL 1795 (holotype)	India	NR_173867	–	–	–	–
*R. langei*	450/BB 07.792	France		KU237510	KU237796	KU237356	Buyck et al. (2018)
*R. lavandula*	RITF6329	Yunnan, China	OR907083	OR907065	OR914534	OR934513	Chen et al. (2024a)
*R. lavandula*	RITF6349	Yunnan, China	OR907084	OR907069	OR914536	OR934514	Chen et al. (2024a)
*R. lavandula*	RITF6340	Yunnan, China	OR907085	OR907066	OR914535	OR934515	Chen et al. (2024a)
*R. lavandula*	RITF3196	Yunnan, China	OR907086	OR907067	–	OR934512	Chen et al. (2024a)
*R. lavandula*	RITF3282 (holotype)	Yunnan, China	OR907087	OR907068	OR914537	OR934511	Chen et al. (2024a)
*R. lilaceofusca*	RITF6330 (holotype)	Yunnan, China	OR907102	OR907075	OR914539	OR934530	Chen et al. (2024a)
*R. lilaceofusca*	RITF2645	Hubei, China	OR907093	OR907074	OR914540	OR934531	Chen et al. (2024a)
*R. lilaceofusca*	RITF3761	Guizhou, China	OR907094	–	–	–	Chen et al. (2024a)
*R. lilaceofusca*	RITF2631	Hubei, China	–	–	OR914541	–	Chen et al. (2024a)
*R. lilacina*	MMCR00191	Thailand	MT940809	MT940819	MT965685	–	Crous et al. (2022)
*R. lotus*	HKAS 79209 (holotype)	Guangdong, China	MG214688	MG214695	–	–	Li and Deng (2018)
*R. lotus*	HKAS 76139	Guangdong, China	MG214687	–	–	–	Li and Deng (2018)
*R. maguanensis*	HKAS 102277 (holotype)	Yunnan, China	MH724918	MH714537	MH939989	–	Wang et al. (2019)
*R. nigrovirens*	HKAS 55222 (holotype)	Yunnan, China	KP171173	–	–	–	Zhao et al. (2015)
*R. nigrovirens*	HKAS 55045	Yunnan, China	KP171175	–	–	–	Zhao et al. (2015)
*R. nigrovirens*	HKAS 69567	Yunnan, China	KP171176	–	–	–	Zhao et al. (2015)
*R. nigrovirens*	HKAS 55042	Yunnan, China	KP171174	–	–	–	Zhao et al. (2015)
*R. nigrovirens*	RITF6408	Yunnan, China	OR907095	OR907073	OR914568	OR934521	Chen et al. (2024a)
*R. pseudocyanoxantha*	CUH AM177 (holotype)	India	NR_173166	–	–	–	Khatua et al. (2021b)
*R. pallidirosea*	UTC 00274382 (holotype)	USA	NR_153259	–	–	–	Kropp (2016)
*R. perviridis*	RITF3131 (holotype)	Yunnan, China	OR907098	OR907072	OR914548	OR934523	Chen et al. (2024a)
*R. perviridis*	RITF2912	Xizang, China	OR907100	OR907070	OR914547	OR934522	Chen et al. (2024a)
*R. perviridis*	RITF6982	Yunnan, China	OR907101	OR907071	OR914545	OR934524	Chen et al. (2024a)
*R. phloginea*	CNX530524068 (holotype)	Yunnan, China	MK860701	MK860704	–	MK860708	Song et al. (2019)
*R. phloginea*	CNX530524304	Yunnan, China	MK860700	MK860703	–	MK860707	Song et al. (2019)
*R. purpureobrunnea*	RITF6289 (holotype)	Yunnan, China	PP700459	–	PP707794	PP700448	Chen et al. (2024b)
*R. purpureobrunnea*	RITF6682	Jiangsu, China	PP700457	PP700467	PP707795	PP700447	Chen et al. (2024b)
*R. purpureobrunnea*	RITF6271	Yunnan, China	PP700456	PP700465	PP707793	PP700446	Chen et al. (2024b)
*R. purpureobrunnea*	RITF7104	Jiangxi, China	PP700458	PP700466	PP707796	PP700449	Chen et al. (2024b)
***R. purpureogrisea***	**HFJAU5628**	Hubei, China	**PZ240600**	**PZ240610**	**PZ268646**	**PZ241899**	**This study**
***R. purpureogrisea***	**HFJAU5642 (holotype)**	Hubei, China	**PZ240601**	**PZ240611**	**PZ268647**	**PZ241900**	**This study**
*R. purpureorosea*	H17050506 (holotype)	Guangdong, China	MK049976	MK881941	–	MK882069	Song (2022)
*R. purpureoviridis*	BBH 49226 (holotype)	Thailand	–	MT940817	MT965684	–	Crous et al. (2022)
***R. subpallescens***	**HFJAU5665 (holotype)**	**Hubei, China**	**PZ240602**	**PZ240612**	**PZ268648**	**PZ241901**	**This study**
*R. subpallidirosea*	K15052818 (holotype)	Guangdong, China	KU863582	MK881923	–	MK882051	Zhang et al. (2017)
*R. substriata*	HKAS 102278 (holotype)	Yunnan, China	MH724921	MH714540	MH939992	–	Wang et al. (2019)
*R. variata*	BPL241	USA	KT933959	KT935818	KT935889	–	Looney et al. (2016)
***R. wuyishanensis***	**HFJAU3581**	**Fujian, China**	**PZ240603**	**PZ240613**	**PZ268649**	**PZ241902**	**This study**
***R. wuyishanensis***	**HFJAU4809 (holotype)**	**Fujian, China**	**PZ240605**	**PZ240615**	**PZ268651**	**PZ241904**	**This study**
***R. wuyishanensis***	**HFJAU4761**	**Fujian, China**	**PZ240604**	**PZ240614**	**PZ268650**	**PZ241903**	**This study**

**Figure 1.**
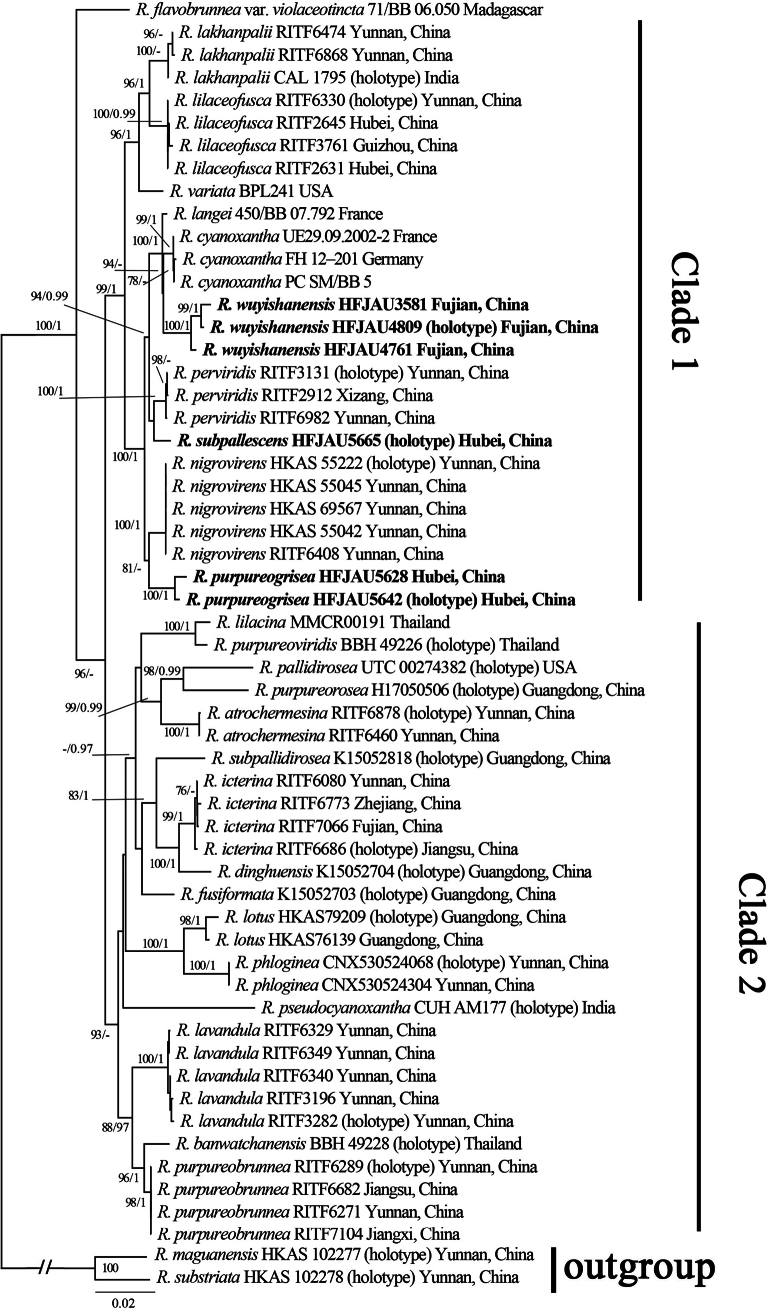
Maximum Likelihood (ML) phylogenetic tree of *Russula* subsect. *Cyanoxanthinae* based on ITS, LSU, mtSSU, and *rpb*2 sequences with *R. maguanensis* and *R. substriata* as outgroups. Maximum Likelihood bootstrap support (UFBoot) ≥ 75 and Bayesian posterior probabilities (BI-PP) ≥ 0.95 are annotated at the nodes as UFBoot / PP. The new species proposed in this paper are bolded.

The best-fit evolutionary models for both BI and ML analyses were determined using ModelFinder (Kalyaanamoorthy et al. 2017) integrated into PhyloSuite (Zhang et al. 2020), based on the Bayesian Information Criterion (BIC). For the BI analysis, four Markov Chain Monte Carlo (MCMC) chains were run for TWO million generations, with trees sampled every 100 generations. The run was conducted for 2,000,000 generations, at which point the average standard deviation of split frequencies dropped below 0.01, indicating full convergence. The first 25% of the sampled trees were discarded as burn-in, and the remaining trees were used to calculate Bayesian posterior probabilities (BI-PP). For the ML analysis, 10,000 replicates were performed based on the ultrafast bootstrap option, which allowed different partitions to have independent evolutionary rates. The branches with Bayesian posterior probability (BI-PP) ≥ 0.95 and ML ultrafast bootstrap proportions (UFBoot) ≥ 95 were considered statistically supported (Nguyen et al. 2015). In the phylogenetic tree (Fig. 1), branches with Bayesian posterior probabilities (BI-PP) ≥ 0.95 and ML ultrafast bootstrap support (UFBoot) ≥ 75 are indicated.

## Results

### Phylogenetic analysis

The phylogenetic analyses included 2,713 characters from 57 taxa (ITS 652 bp; LSU 890 bp; mtSSU 508 bp; *rpb*2 663 bp), comprising 2,201 constant sites, 372 parsimony-informative sites, and 140 singleton sites. The best-fit evolutionary models selected for both Maximum Likelihood (ML) and Bayesian Inference (BI) analyses were K2P+G4 for ITS and *rpb*2, K2P+I+G4 for LSU, and F81+F+I for mtSSU.

The results of the phylogenetic analyses are shown in Fig. 1. All three new species described in this study belong to Clade 1 (Chen et al. 2024a) and each formed an independent branch with stable statistical support. The new species *Russula wuyishanensis* formed an independent lineage (UFBoot = 100%, BI-PP = 1) and grouped with *R. cyanoxantha* (Schaeff.) Fr. and *R. langei* Bon (UFBoot = 100%, BI-PP = 1). The new species *Russula subpallescens* grouped with *R. perviridis* Y.L. Chen, B. Chen & J.F. Liang, and formed a clade with unstable statistical support (UFBoot < 95%, BI-PP < 0.95). The new species *Russula purpureogrisea* formed a distinct and stable lineage (UFBoot = 100%, BI-PP = 1) and showed a close phylogenetic affinity to *R. nigrovirens* Q. Zhao, Y.K. Li & J.F. Liang, although the support for this larger grouping was unstable in the Bayesian analysis (UFBoot = 81%, BI-PP < 0.95).

### Taxonomy

#### Russula purpureogrisea

Taxon classificationFungiRussulalesRussulaceae

J.Q. Yan, B.J. Zhong & S.N. Wang sp. nov.

360146DD-931E-5816-94A6-FA2975F40BCA

863209

Fig. 2

##### Diagnosis.

*Russula purpureogrisea* is mainly characterised by its small to medium-sized basidiomata, a dark greyish-ruby to greyish-brown pileus, unforked and rather crowded lamellae with 14–15 per cm at the margin and lamellulae 1/5 to 3/4 of the lamellae length, basidiospores with ornamentation composed mostly of obtuse-conical isolated warts, occasionally connected by fine lines or low ridges, occasionally forming a subreticulum locally, subcylindrical to clavate pleurocystidia with obtuse or acute apices and frequent papillae, subcylindrical to clavate cheilocystidia with obtuse or acute apices and occasional papillae, and cylindrical or clavate pileocystidia with frequent papillae.

**Figure 2.**
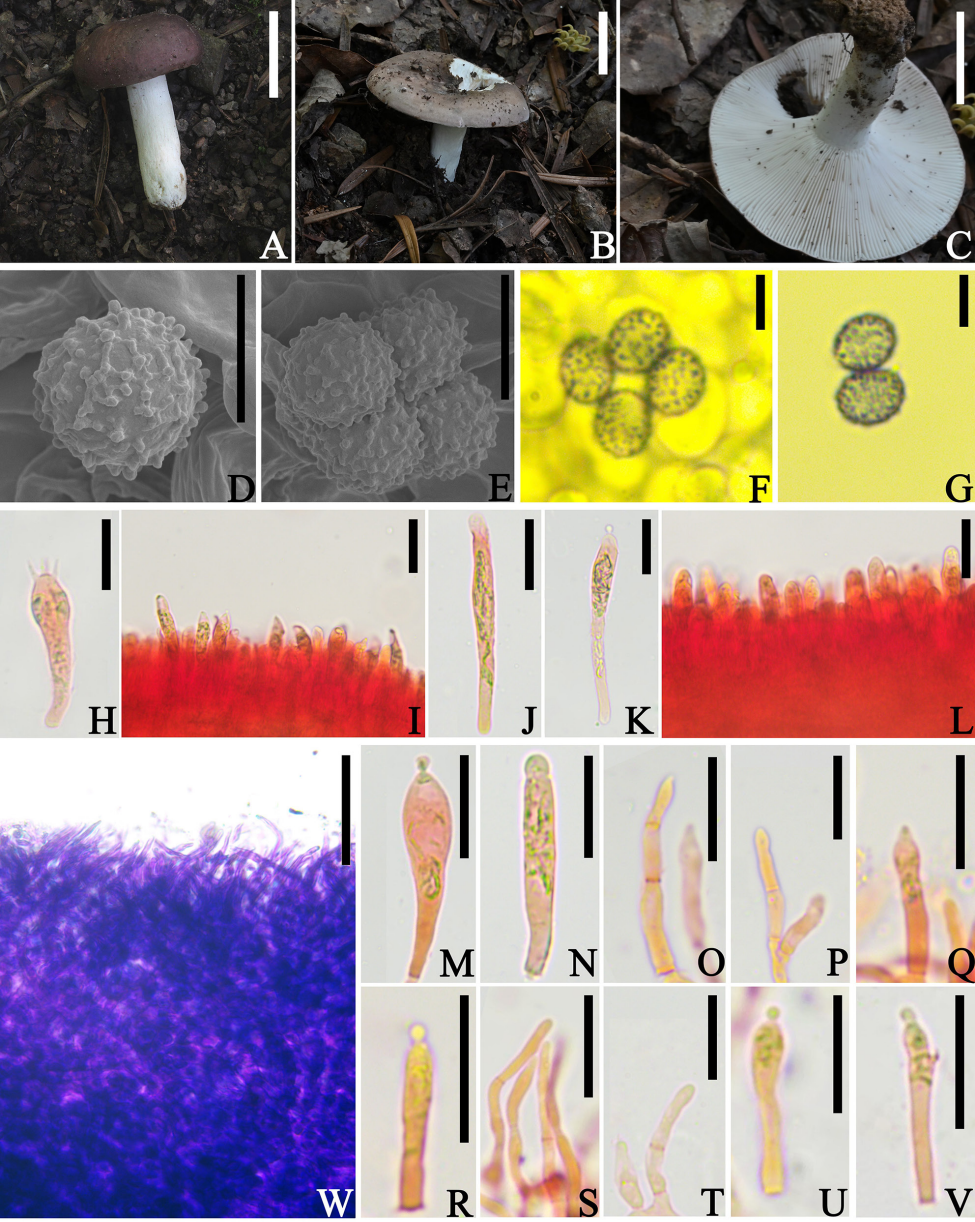
*Russula purpureogrisea*. **A–C**. Basidiomata; **D–G**. Basidiospores; **H**. Basidia; **I–K**. Pleurocystidia; **L–N**. Cheilocystidia; **O, P**. Hyphal terminations near the pileus margin; **Q, R**. Pileocystidia near the pileus margin; **S, T**. Hyphal terminations near the pileus centre; **U, V**. Pileocystidia near the pileus centre; **W**. Pileipellis. **F–G**. In Melzer’s reagent; **H–V**. In 5% KOH stained with 1% Congo red; **W**. In water after staining with 1% cresyl blue. Scale bars: 20 mm (**A–C**); 5 μm (**D–G**); 20 μm (**H–V**); 50 μm (**W**).

##### Holotype.

China • Hubei Province, Yichang City, Xingshan County, Heyuan, 31°28'10"N, 110°50'49"E, alt. 491 m, 23 Jul 2024, Jun-Qing Yan, Lin-Gen Chen, Ling Ding and Hong Chen (HFJAU5642).

##### Etymology.

‘purpureogrisea’ refers to a purplish-gray pileus.

##### Chinese name.

紫灰红菇.

##### Description.

***Basidiomata*** small to medium-sized. Pileus 20–50 mm in diameter, hemispherical when young, plano-convex and depressed in the centre when mature; surface dry, smooth, glabrous; dark greyish ruby (12E3) when young, becoming greyish brown (9F3) at the centre in age, paler towards the margin. ***Lamellae*** 1.5–3.0 mm wide, sinuate, without furcations, rather crowded, 14–15 per cm near pileus margin; lamellulae present, 1/5 to 3/4 of the lamellae length, white (1A1). ***Stipe*** 26–37 × 7.5–10.0 mm, cylindrical, central, solid, white (1A1). ***Context*** thick, white (1A1), unchanging when bruised; taste and odour not recorded.

***Basidiospores*** (6.1–)6.6–8.1(–8.4) × (5.2–)5.3–6.6(–6.8) μm, Q = (1.01–)1.14–1.29(–1.30), globose, subglobose or broadly ellipsoid; ornamentation composed mostly of obtuse-conical isolated warts, moderately dense (3–5 in a 3 μm diam. circle), occasionally connected by fine lines or low ridges, occasionally forming a subreticulum locally, warts 0.2–0.6 μm high. ***Basidia*** 39.5–49.5 × 10.0–12.5 μm, clavate, 2- or 4-spored. ***Hymenial cystidia on lamellae sides*** 47.0–76.0 × 6.5–10.0 μm, subcylindrical to clavate, apically obtuse or acute, often with a papilla, with contents, thin-walled, contents heteromorphous, turning greyish-black in sulphovanillin. ***Hymenial cystidia on lamellae edges*** 39.5–55.5 × 5.5–9.5 μm, subcylindrical to clavate, apically obtuse or acute, with occasional papillae, contents heteromorphous, turning greyish-black in sulphovanillin. ***Pileipellis*** metachromatic in cresyl blue, distinctly two-layered, ca. 120–190 μm thick; suprapellis 50–75 μm deep, composed of slender, erect to repent hyphae that are attenuated at the apex; subpellis 70–115 μm deep, composed of interwoven hyphae. Hyphal terminations near the pileus margin thin-walled, often flexuous, sometimes forked; terminal cells 9.5–22.5 × 2.0–4.0 μm, subcylindrical, apically attenuated or constricted. Hyphal terminations near the pileus centre similar to those near the margin; terminal cells 12.5–23.5 × 2.0–3.5 μm, subcylindrical, apically obtuse or attenuated. ***Pileocystidia*** near the pileus margin 18.5–31.5 × 3.0–6.5 μm, clavate or cylindrical, apically obtuse or acute, often with a papilla, thin-walled; contents granular, turning greyish-black in sulphovanillin. Pileocystidia near the pileus centre 18.5–33.5 × 3.0–4.5 μm, clavate or cylindrical, apically obtuse or acute, often with a papilla, thin-walled; contents granular, turning greyish-black in sulphovanillin. ***Cystidioid hyphae*** in pileipellis and context with heteromorphous or crystalline contents; oleiferous hyphae in subpellis with refractive contents.

##### Habitat.

Mixed coniferous and broad-leaved forest dominated by Cupressaceae, Pinaceae, Theaceae, and Fagaceae.

##### Additional specimens examined.

China • Hubei Province: Yichang City, Xingshan County, Heyuan, 31°28'10"N, 110°50'49"E, alt. 491 m, 23 July 2024, Jun-Qing Yan, Lin-Gen Chen, Ling Ding and Hong Chen, HFJAU5628.

##### Notes.

Based on morphological characteristics such as small to medium-sized basidiomata, a dark greyish-ruby to greyish-brown pileus, the presence of lamellulae, basidiospores with an inamyloid suprahilar plage, a pileipellis that is metachromatic in cresyl blue, and the presence of unicellular pileocystidia, this species is assigned to subgenus *Heterophyllidiae* subsection *Cyanoxanthinae*. Phylogenetically, this species shares the highest ITS sequence similarity with *R. perviridis* (95%), *R. nigrovirens* (94%), and *R. cyanoxantha* (93%). It also shares LSU sequence similarities in the range of 96%–98% with *R. variata* Banning, *R. parvisaxoides* (T. Lebel) T. Lebel, and *R. banwatchanensis* Sommai. However, *R. perviridis* has a greyish-green to dark green pileus, lamellae turning yellowish-brown when bruised, and longer pileocystidia (36.5–60.0 μm) (Chen et al. 2024a). *Russula nigrovirens* has a dark green pileus, wider basidiospores (up to 8 μm), and longer pileocystidia (up to 62.0 μm) (Zhao et al. 2015). *Russula cyanoxantha* has a greenish-purple pileus, larger basidiomata (pileus diameter up to 12 cm), and longer hymenial cystidia (up to 100 μm) (Bon 1988; Sarnari 1998). *Russula variata* features lamellae turning rust-brown when bruised, spore ornamentation arranged in chains and connected into ridges, and narrower basidia (7–8.5 µm) (Sarnari 1998). *Russula parvisaxoides* forms sequestrate basidiomata with a white surface bearing pale yellow to orange-brown patches, and longer basidiospores (up to 11 μm) (Lebel 2002). *Russula banwatchanensis* has longitudinal striations on the stipe, lower spore ornamentation (not exceeding 1.0–2.0 μm), and longer pileocystidia near the margin (42.5–127.5 μm) (Crous et al. 2022).

Morphologically, only *R. brunneoviolacea* Crawshay, *R. phloginea*, and *R. pseudocyanoxantha* Paloi are quite similar to this species, as all share purplish pileus tones, basidiospore sizes ranging from 6.0–9.0 × 5.0–7.0 μm, and subcylindrical or clavate hymenial cystidia. However, *R. brunneoviolacea* has a stipe that turns yellow locally when bruised, higher spore ornamentation (1–1.6 μm), and wider pileocystidia (up to 9 μm) (Sarnari 1998). *Russula phloginea* features small white crust-like warts or saddle-brown thin scales on the pileus and longer pileocystidia (up to 56 μm) (Song et al. 2019). *Russula pseudocyanoxantha* has a hollow stipe that turns pale yellow when bruised, and spore ornamentation composed of completely isolated warts without any connections (Khatua et al. 2021b).

#### Russula subpallescens

Taxon classificationFungiRussulalesRussulaceae

J.Q. Yan, B.J. Zhong & S.N. Wang sp. nov.

12A5FCF6-92B4-564B-B45B-6E2ABFDACBE3

863214

Fig. 3

##### Diagnosis.

*Russula subpallescens* is mainly characterised by its medium-sized basidiomata, a pinkish-white pileus, adnate, unforked, and crowded lamellae of equal length with 10–13 per cm at the margin and intervenose veins, and a white stipe that develops pale yellow to maize yellow patches when old or bruised for a long time; basidiospores with ornamentation composed of dispersed warts, frequently connected by short lines, nearly subreticulate, subcylindrical to fusiform hymenial cystidia with obtuse or acute apices and frequent papillae, and cylindrical pileocystidia with obtuse or attenuated apices and papillae.

**Figure 3.**
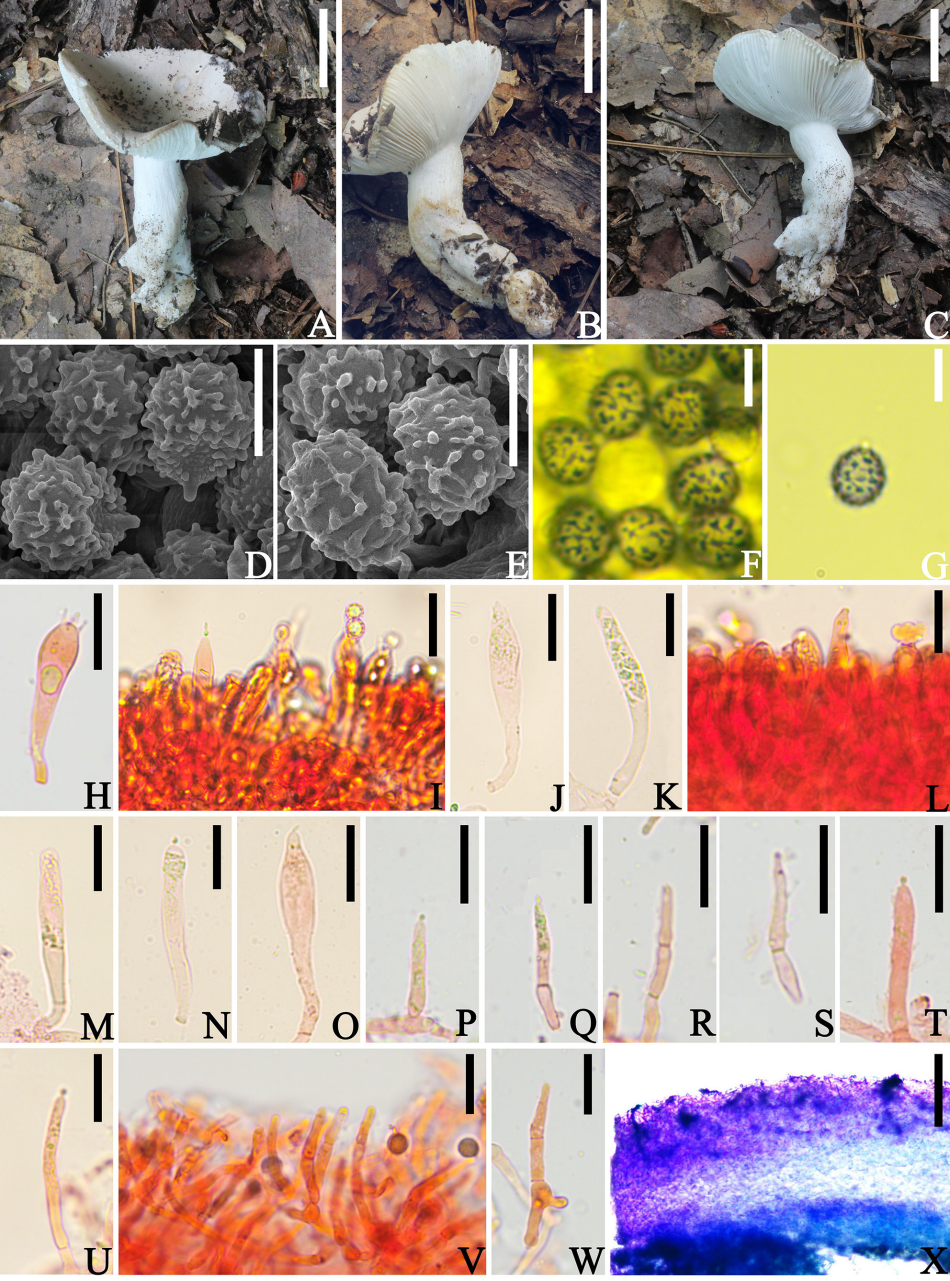
*Russula subpallescens*. **A–C**. Basidiomata; **D–G**. Basidiospores; **H**. Basidia; **I–K**. Pleurocystidia; **L–O**. Cheilocystidia; **P, Q**. Pileocystidia near the pileus centre; **R, S**. Hyphal terminations near the pileus centre; **T, U**. Pileocystidia near the pileus margin; **V, W**. Hyphal terminations near the pileus margin; **X**. Pileipellis. **F–G**. In Melzer’s reagent; **H–W**. In 5% KOH stained with 1% Congo red; **X**. In water after staining with 1% cresyl blue. Scale bars: 20 mm (**A–C**); 5 μm (**D–G**); 20 μm (**H–W**); 100 μm (**X**).

##### Holotype.

China • Hubei Province, Yichang City, Xingshan County, Longdongpo, 31°23'47"N, 110°55'48"E, alt. 1526 m, 24 Jul 2024, Jun-Qing Yan, Lin-Gen Chen, Hong Chen, Ling Ding (HFJAU5665).

##### Etymology.

‘subpallescens’ refers to its morphology similar to ‘*Russula pallescens*’.

##### Chinese name.

近粉白红菇.

##### Description.

***Basidiomata*** medium-sized. Pileus ca. 55 mm in diameter, shallowly infundibuliform; surface smooth, dry, glabrous, pinkish-white (10A2). ***Lamellae*** 2.0–3.5 mm wide, adnate, without furcations, equal in length, crowded, 10–13 per cm near pileus margin, with intervenose veins, white (1A1). ***Stipe*** ca. 41 × 15 mm, cylindrical, central, white (1A1), hollow, but developing pale yellow (4A4) to maize yellow (4B5) patches when old or long after being bruised. ***Context*** thick, white (1A1), unchanging when bruised; taste and odour not recorded.

***Basidiospores*** (6.7–)6.9–7.9(–8.4) × (5.8–)5.9–6.7(–6.8) μm, Q = (1.03–)1.11–1.26(–1.32), globose, subglobose, broadly ellipsoid or ellipsoid; ornamentation composed of many dispersed, obtuse-conical to subcylindrical amyloid warts, moderately dense (3–7 in a 3 μm diam. circle), frequently connected by short lines, nearly subreticulate, 0.3–0.8 μm high; suprahilar plage inamyloid. ***Basidia*** 39.5–52.0 × 8.5–11.5 μm, clavate, 2- or 4-spored. ***Hymenial cystidia on lamellae sides*** 50.5–74.0 × 7.0–11.5 μm, subcylindrical or fusiform, apically obtuse or acute, often with a papilla, thin-walled, contents granulose, turning greyish-black in sulphovanillin. ***Hymenial cystidia on lamellae edges*** 53.0–66.5 × 5.0–9.0 μm, subcylindrical or fusiform, apically obtuse or acute, often with a papilla, contents granulose, turning greyish-black in sulphovanillin. ***Pileipellis*** metachromatic in cresyl blue, indistinctly two-layered, ca. 120–250 μm thick; suprapellis 60–90 μm deep, composed of erect or ascending hyphae that are attenuated at the apex; subpellis 60–150 μm deep, composed of interwoven hyphae. Hyphal terminations near the pileus margin thin-walled, occasionally flexuous; terminal cells 11.0–28.0 × 2.5–5.0 μm, cylindrical, apically attenuated or obtuse. Hyphal terminations near the pileus centre similar to those near the margin; terminal cells 9.5–24.5 × 2.5–4.5 μm, cylindrical, apically attenuated or obtuse. ***Pileocystidia*** near the pileus margin 20.0–56.5 × 4.0–6.0 μm, cylindrical, apically obtuse or attenuated, with a papilla, thin-walled; contents granular, turning greyish-black in sulphovanillin. Pileocystidia near the pileus centre 17.0–44.5 × 3.5–5.0 μm, cylindrical, apically obtuse or attenuated, with a papilla, thin-walled; contents granular, turning greyish-black in sulphovanillin. ***Cystidioid hyphae*** in pileipellis and context with heteromorphous contents; oleiferous hyphae in subpellis with refractive contents.

##### Habitat.

Mixed coniferous and broad-leaved forest dominated by Cupressaceae, Pinaceae, Theaceae, and Fagaceae.

##### Notes.

Based on the medium-sized basidiomata, pinkish-white pileus, basidiospores with an inamyloid suprahilar plage, metachromatic reaction of the pileipellis in cresyl blue, and unicellular pileocystidia, this species can be classified into *Russula* subg. *Heterophyllidiae* subsect. *Cyanoxanthinae*. Within this section, *R. subpallescens* shares ITS sequence similarities in the range of 97%–98% with *R. cyanoxantha* (Schaeff.) Fr., *R. nigrovirens* Q. Zhao, Y.K. Li & J.F. Liang, and *R. perviridis* Y.L. Chen, B. Chen & J.F. Liang. However, *R. cyanoxantha* has spore ornamentation composed of isolated warts without connecting lines and longer pleurocystidia (up to 100 µm) (Bon 1988; Sarnari 1998). *Russula nigrovirens* has a greenish-white to greyish-green pileus with patches, rare lamellae furcations only near the stipe, and shorter cheilocystidia (46.0–55.0 µm) (Zhao et al. 2015). *Russula perviridis* differs by having a greyish-green to dark green pileus (sometimes tinged yellowish-brown), reticulate spore ornamentation, and frequently forked lamellae (Chen et al. 2024a).

Morphologically, *R. subpallidirosea* J.B. Zhang & L.H. Qiu, *R. vesca* Fr., and *R. galochroa* (Fr.) Fr. are quite similar to this species, as all share white to pinkish pileus tones, white stipe with yellow to brown spots, and basidiospores ranging from 6.5–8.5 × 5.5–7.0 μm. However, *R. subpallidirosea* can be distinguished by its frequently forked lamellae, relatively lower spore ornamentation (< 0.6 μm), and shorter pleurocystidia (35–50 × 5–8 μm) (Zhang et al. 2017). *Russula vesca* has spore ornamentation composed of isolated warts with a lower height (< 0.5 μm) and lacks pileocystidia (Sarnari 1998). *Russula galochroa* features extremely low spore ornamentation (0.1–0.2 μm) that is completely isolated without any connections (Knudsen and Vesterholt 2012).

#### Russula wuyishanensis

Taxon classificationFungiRussulalesRussulaceae

J.Q. Yan, B.J. Zhong & S.N. Wang sp. nov.

67D9D92C-DB55-57AC-A188-4CD390BC7616

863215

Fig. 4

##### Diagnosis.

*Russula wuyishanensis* is mainly characterised by its small to medium-sized basidiomata, a reddish-grey to purplish-brown pileus with a slightly greyish to dark greyish-green centre, three types of lamellulae, basidiospores with many obtuse isolated warts partially connected by short lines or ridges and occasionally subreticulate, clavate, subcylindrical or fusiform hymenial cystidia with obtuse or acute apices and frequent papillae, and cylindrical or clavate pileocystidia with obtuse or acute apices and frequent papillae.

**Figure 4.**
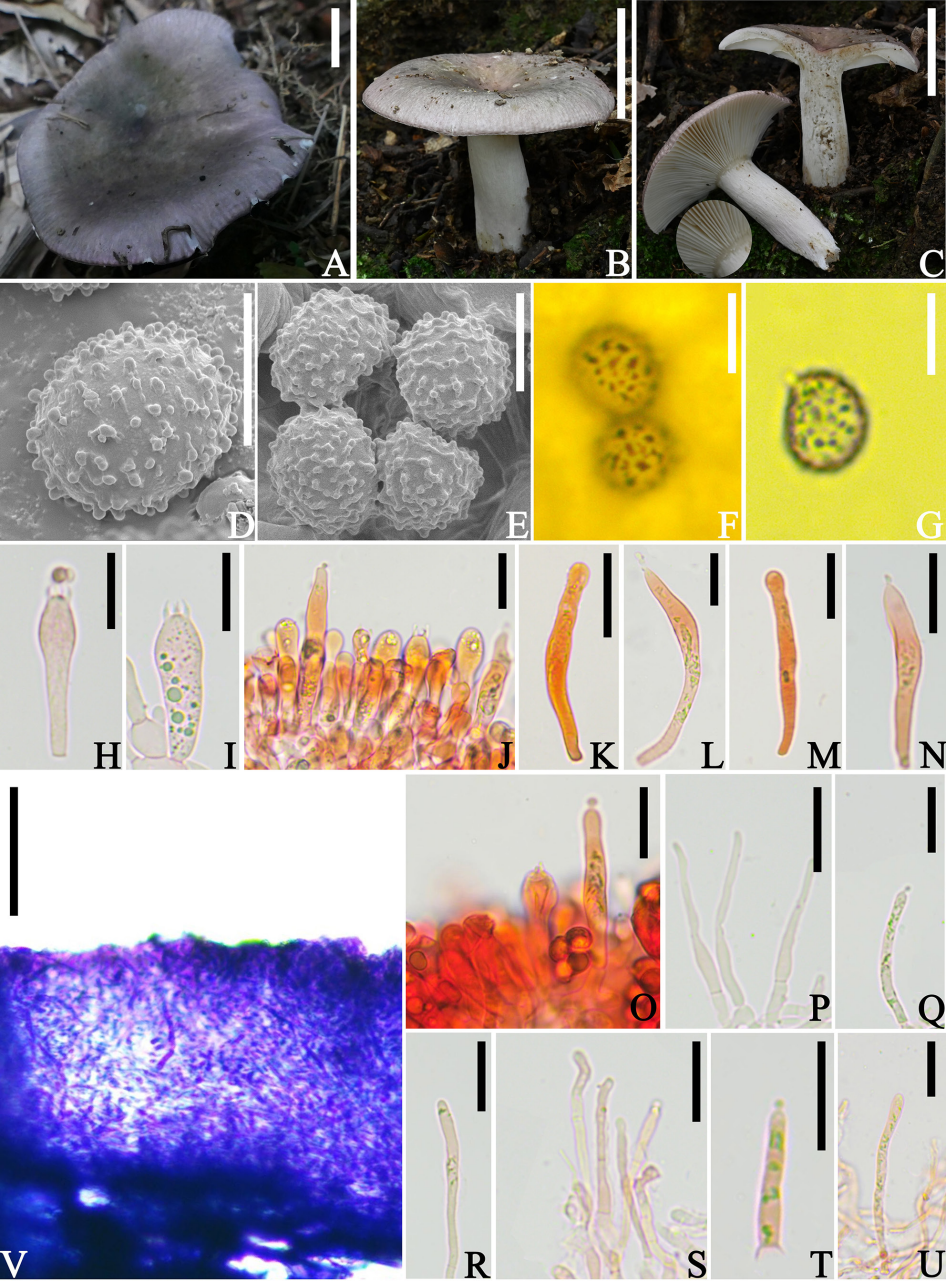
*Russula wuyishanensis*. **A–C**. Basidiomata; **D–G**. Basidiospores; **H, I**. Basidia; **J–L**. Pleurocystidia; **M–O**. Cheilocystidia; **P**. Hyphal terminations near the pileus margin; **Q, R**. Pileocystidia near the pileus margin; **S**. Hyphal terminations near the pileus centre; **T, U**. Pileocystidia near the pileus centre; **V**. Pileipellis. **F–G**. In Melzer’s reagent; **H–U**. In 5% KOH stained with 1% Congo red; **V**. In water after staining with 1% cresyl blue. Scale bars: 20 mm (**A–C**); 5 μm (**D–G**); 20 μm (**H–U**); 50 μm (**V**).

##### Holotype.

China • Fujian Province, Nanping City, Shaowu City, Wuyishan Longhu Logging Camp, 27°32'16"N, 117°30'47"E, alt. 478 m, 9 May 2023, Hui Zeng, Zhi-Heng Zeng, Bin-Rong Ke, Cheng-Feng Nie (HFJAU4809).

##### Etymology.

‘wuyishanensis’ refers to Wuyishan, the type locality of the species.

##### Chinese name.

武夷山红菇.

##### Description.

***Basidiomata*** small to medium-sized. Pileus 45–75 mm in diameter, applanate to depressed in the centre; surface slightly viscid, smooth, glabrous, occasionally cracked; young pileus reddish-grey (11B2), centre slightly greyish (11F1), mature pileus purplish-brown (11E4) with a darker centre or tinged dark greyish-green (26F3). ***Lamellae*** 3.0–4.0 mm wide, decurrent, without furcations, with 3 types of lamellulae, moderately crowded, 8–11 per cm near pileus margin, white (1A1), occasionally with rust-colored (6E8) patches. ***Stipe*** 35–45 × 7.0–9.0 mm, cylindrical, central, spongy, white (1A1). ***Context*** thick, white (1A1), turning yellowish-brown (5D4) when bruised; taste and odour not recorded.

***Basidiospores*** (6.2–)7.0–8.9(–9.7) × (5.2–)6.0–7.2(–7.7) μm, Q = (1.08–)1.13–1.35(–1.40), globose, subglobose, broadly ellipsoid or ellipsoid; ornamentation composed of many obtuse isolated warts, sparse (2–5 in a 3 μm diam. circle), partially connected by short lines or ridges, occasionally subreticulate, amyloid; suprahilar plage inamyloid; warts 0.3–0.6 μm high. ***Basidia*** 38.0–54.0 × 9.5–11.5 μm, clavate or subclavate, 2- or 4-spored. ***Hymenial cystidia on lamellae sides*** 48.5–87.5 × 6.0–8.5 μm, clavate, subcylindrical or fusiform, apically obtuse or acute, often with a papilla, thin-walled, contents heteromorphous or crystalline, turning greyish-black in sulphovanillin. ***Hymenial cystidia on lamellae edges*** 43.5–75.0 × 5.0–8.0 μm, clavate, subcylindrical or fusiform, apically obtuse or acute, often with a papilla, contents heteromorphous, turning greyish-black in sulphovanillin. ***Pileipellis*** metachromatic in cresyl blue, indistinctly two-layered, ca. 150–210 μm thick; suprapellis 60–90 μm deep, composed of erect to repent hyphae that are attenuated at the apex; subpellis 60–120 μm deep, composed of interwoven hyphae. Hyphal terminations near the pileus margin thin-walled, often flexuous, sometimes forked; terminal cells 15.0–39.5 × 2.5–4.0 μm, subcylindrical, apically attenuated or constricted. Hyphal terminations near the pileus centre similar to those near the margin; terminal cells 15.2–41.4 × 2.2–3.0 μm, subcylindrical, apically attenuated or constricted. ***Pileocystidia*** near the pileus margin 18.0–72.5 × 2.5–4.5 μm, cylindrical or clavate, apically obtuse or acute, often with a papilla, thin-walled; contents granular, turning greyish-black in sulphovanillin. Pileocystidia near the pileus centre 16.0–81.5 × 3.0–5.0 μm, cylindrical or clavate, apically obtuse or acute, often with a papilla, thin-walled; contents granular, turning greyish-black in sulphovanillin. ***Cystidioid hyphae*** in pileipellis and context with granulose or heteromorphous contents; oleiferous hyphae in subpellis with refractive contents.

##### Habitat.

Mixed coniferous and broad-leaved forest dominated by Cupressaceae, Pinaceae, Theaceae, and Fagaceae.

##### Additional specimens examined.

China • Fujian Province: Nanping City, Guangze County, Wuyishan, Lingxiaxi, 27°32'39"N, 117°28'09"E, alt. 402 m, 7 June 2022, Jun-Qing Yan and Lin-Gen Chen, HFJAU3581; • Nanping City, Shaowu City, Wuyishan, Longhu Logging Camp, 27°32'16"N, 117°30'47"E, alt. 478 m, 9 May 2023, Hui Zeng, Zhi-Heng Zeng, Bin-Rong Ke and Cheng-Feng Nie, HFJAU4761.

##### Notes.

Based on characteristics such as small to medium-sized basidiomata, a reddish-grey to purplish-brown pileus, the presence of lamellulae, basidiospores with an inamyloid suprahilar plage, a pileipellis that is metachromatic in cresyl blue, and the presence of unicellular pileocystidia, this species is assigned to subgenus *Heterophyllidiae* subsection *Cyanoxanthinae*. Phylogenetically, this species shares the highest ITS sequence similarity (95%–97%) with *R. perviridis* Y.L. Chen, B. Chen & J.F. Liang, *R. cyanoxantha* (Schaeff.) Fr., and *R. nigrovirens* Q. Zhao, Y.K. Li & J.F. Liang. Its LSU sequence shares similarities in the range of 96%–98% with *R. lotus* F. Li, *R. sublaevis* (Buyck) Buyck, and *R. vesca* Fr. However, *R. perviridis* has a greyish-green to dark green pileus, frequently forked lamellae, and partially reticulate spore ornamentation (Chen et al. 2024a). *Russula cyanoxantha* has a greenish-purple pileus and longer cylindrical hymenial cystidia (up to 100 μm) (Bon 1988; Sarnari 1998). *Russula nigrovirens* has a dark green pileus with the surface cracking into small patches and shorter cheilocystidia (up to 55.0 μm) (Zhao et al. 2015). *Russula lotus* features a pinkish pileus, completely isolated spore warts without connections, and wider pleurocystidia (up to 16.0 μm) (Li and Deng 2018). *Russula sublaevis* has a yellow pileus, nearly smooth spore ornamentation, and wider pleurocystidia (up to 13.0 μm) (Manz et al. 2025). *Russula vesca* has a pale purplish-flesh or wine-brown pileus, forked lamellae, and subfusiform or subclavate pileocystidia (Sarnari 1998).

Morphologically, only *R. purpureorosea* Yu Song, *R. phloginea* J. Song & Jun F. Liang, and *R. lavandula* Y.L. Chen are quite similar to this species, as all share purplish pileus tones, spore ornamentation height not exceeding 0.6 μm, and clavate or fusiform hymenial cystidia. However, *R. purpureorosea* has intervenose lamellae, longer cheilocystidia (up to 95 μm), and shorter terminal cells at the pileus centre (6.5–15.5 μm) (Song 2022). *Russula phloginea* features small white crust-like spots or saddle-brown thin scales on the pileus surface, frequently forked lamellae near the stipe, and often moniliform pileocystidia (Song et al. 2019). *Russula lavandula* has a yellow centre on the pileus, a pale-yellow tinge at the stipe base, and shorter hymenial cystidia (up to 64.5 μm) (Chen et al. 2024a).

## Discussion

The multi-locus phylogenetic analyses in this study strongly corroborate the phylogenetic results proposed by Chen et al. (2024a, 2024b). In the phylogenetic tree (Fig. 1), the three novel species discovered herein are unambiguously nested within Clade 1 of subsect. *Cyanoxanthinae*. Specifically, Chen et al. (2024a) proposed that subsect. *Cyanoxanthinae* could be divided into two major clades, noting that they can be distinguished by the connectivity of their basidiospore ornamentation: Clade 1 is characterized by having more connections between spore warts, whereas Clade 2 features predominantly isolated warts. In the present study, the basidiospore ornamentation of *Russula subpallescens* (frequently connected by short lines) closely aligns with the definition of Clade 1.

Notably, although *R. purpureogrisea* and *R. wuyishanensis* consistently cluster within Clade 1 in the molecular phylogeny, their basidiospore ornamentations predominantly consist of isolated warts, only partially or occasionally connected by fine lines and short ridges. This morphological variation directly addresses the inference by Chen et al. (2024a) that “further studies are needed to determine the critical point of the number or density of connections,” suggesting that the morphological boundary between these two clades is likely more complex than previously assumed. Future studies incorporating broader geographic sampling are required to elucidate the evolutionary history and ecological characteristics of this subsection, thereby further deepening our understanding of its species diversity.

### Key to known species in *R.* subsect. *Cyanoxanthinae* in China

**Table:** 

1	Lamellulae absent or rare	**2**
–	Lamellulae irregularly inserted, but common (more frequent than usual for subg. *Heterophyllidiae*)	**4**
2	Reaction of hymenial cystidia to SV negative	***R. fusiformata***
–	Reaction of hymenial cystidia to SV positive	**3**
3	Pileus greyish green to yellowish green; pileocystidia at the margin epapillate (without papillae)	***R. icterina***
–	Pileus pinkish white; pileocystidia at the margin papillate	***R. subpallescens***
4	Basidiospore ornamentation mainly subreticulate to reticulate	**5**
–	Basidiospore ornamentation mainly composed isolated	**6**
5	Lamellae extremely crowded (20–24 /cm at pileus margin)	***R. lilaceofusca***
–	Lamellae moderately crowded (9–12 /cm at pileus margin)	***R. perviridis***
6	Lamellar furcations extremely rare, nearly absent	**7**
–	Lamellar furcations irregularly inserted, but common (more frequent than usual for subg. *Heterophyllidiae*)	**12**
7	Pileus with green tints	**8**
–	Pileus without green tints	**10**
8	Context discoloring (yellowish brown) when bruised	***R. wuyishanensis***
–	Context unchanging when bruised	**9**
9	Occurring at high elevations (> 3000 m a.s.l.); basidia relatively long (up to 75 μm)	***R. nigrovirens***
–	Occurring at low elevations (< 1500 m a.s.l.); basidia relatively short (≤ 55 μm)	***R. dinghuensis***
10	Basidiospore ornamentation relatively high (often > 0.6 μm, up to 1.5–2.0 μm)	***R. lotus***
–	Basidiospore ornamentation relatively low (usually ≤ 0.6 μm)	**11**
11	Lamellae adnate; reaction of pleurocystidia to SV negative	***R. purpureorosea***
–	Lamellae sinuate; reaction of pleurocystidia to SV positive	***R. purpureogrisea***
12	Reaction of hymenial cystidia to SV yellow	***R. atrochermesina***
–	Reaction of hymenial cystidia to SV not yellow (reddish black, greyish black, or grey)	**13**
13	Reaction of hymenial cystidia to SV reddish black	***R. lavandula***
–	Reaction of hymenial cystidia to SV grey or greyish black	**14**
14	Pileipellis often with purplish brown patches; SV reaction greyish black	***R. purpureobrunnea***
–	Pileipellis without purplish brown patches; SV reaction grey	**15**
15	Lamellae sinuate; pleurocystidia relatively long (up to 80 μm); apices of pileocystidia often moniliform	***R. phloginea***
–	Lamellae adnate; pleurocystidia relatively short (up to 50 μm); apices of pileocystidia not moniliform (mucronate or subapically constricted)	***R. subpallidirosea***

## Supplementary Material

XML Treatment for Russula purpureogrisea

XML Treatment for Russula subpallescens

XML Treatment for Russula wuyishanensis

## Additional information

### Artificial Intelligence (AI) use

The authors accept full responsibility for the content of the manuscript, including the disclosure of any use of AI.

No AI tools were used in the preparation of this manuscript.

### Author ORCIDs

B.-J. Zhong https://orcid.org/0009-0004-0817-9161

H. Zeng https://orcid.org/0000-0003-2025-844X

S.-N. Wang https://orcid.org/0000-0003-0648-271X

J.-Q. Yan https://orcid.org/0000-0003-1128-5171

### Data availability

All of the data that support the findings of this study are available in the main text.
